# Supplementary material for: Improving HIV stigma in the marginalized population in Khorramabad, Iran: A single-blinded randomized, controlled educational trial using role-playing and lecturing
Source: PLOS Glob Public Health. 2023 Mar 7;3(3):e0000689. doi: 10.1371/journal.pgph.0000689 (PMC10022175; doi:10.1371/journal.pgph.0000689)
Supplement: S1 File — (PDF) [file pgph.0000689.s001.pdf]

Clinical Trial Protocol
Iranian Registry of Clinical Trials

26 Oct 2021

Comparison of the effect of role playingmethod with lecture on knowledge and attitude toward HIV/AIDS in general population (18-49 years old) of Khorramabad countryside

Protocol summary

Study aim

Assessing the effect of Role Playing and lecture methods on HIV-related knowledge and attitude among 18-49 years olds marginal population in Khorramabad

Design

270 people. Falkaldin region: role-playing method; stack region: the lecture method; Massor area: control group. The clinical trial is phase 3

Settings and conduct

270 people aged 18-49 years from the general population of the suburbs in Rasht and Falkaddin areas of Khorramabad and Masur residents are considered as the control population, using random sampling. Class and selection criteria will be selected and included in the study. Three suburban areas will be allocated to intervention and control groups by cluster randomization method. In the Falkadine area, the role-playing method is considered to be the lecture method in the Poshteh area. A questionnaire for all individuals will be completed prior to the intervention. Interventions are grouped and the training program will be held once a week, once a week. In order to better study the groups, the groups will be broken down into 20-person subgroups, with 4 one-hour training sessions in each group. After two weeks, the questionnaire will be completed again for all individuals in all three groups. Finally, the information collected before and after the intervention will be analyzed.

Participants/Inclusion and exclusion criteria

Inclusion criteria: • 18-49 years old • Iranian citizenship • A resident of Falkodin, Ridge, and Masur areas of Khorramabad • literate • Mental ability to understand training and answer questions • informed consent to participate Exclusion criteria: • unwillingness to participate in the study • Excluded from the study due to immigration or death

Intervention groups

Teaching by lecture; Role-Playing Training

Main outcome variables

Knowledge and attitude towards HIV

General information

Reason for update

Acronym

IRCT registration information

IRCT registration number: **IRCT20190807044467N1**
Registration date: **2019-12-15, 1398/09/24**
Registration timing: **prospective**

Last update: **2019-12-15, 1398/09/24**
Update count: **0**

Registration date

2019-12-15, 1398/09/24

Registrant information

Name

maryam nasirian

Name of organization / entity

Country

Iran (Islamic Republic of)

Phone

+98 31 3792 3246

Email address

maryamnasirian17@gmail.com

Recruitment status

Recruitment complete

Funding source

Expected recruitment start date

2019-12-22, 1398/10/01

Expected recruitment end date

2020-02-20, 1398/12/01

Actual recruitment start date

empty

Actual recruitment end date

empty

Trial completion date

empty

Scientific title

Comparison of the effect of role playingmethod with lecture on knowledge and attitude toward HIV/AIDS in general population (18-49 years old) of Khorramabad countryside

Public title

The impact of role playing and speaking on population knowledge and attitude

Purpose

Education/Guidance

Inclusion/Exclusion criteria

Inclusion criteria:

Elementary Literacy Mental ability to understand education and answer question Resident in the study areas informed consent to participate in the study

Exclusion criteria:

Under 18 year Over 49 year Unwilingness to participate in the study Exclusion due to immigration or death Knownntal mental disorders and severe visual and auditory disorders

Age

From **18 years** old to **49 years** old

Gender

Both

Phase

N/A

Groups that have been masked

- Participant

Sample size

Target sample size: **270**

Randomization (investigator's opinion)

Randomized

Randomization description

Cluster random allocation; The three regions of Poshteh, Masour, and Falkodin in Khorramabad will be considered as clusters and assigned randomly to intervention group 1 (lecture = Falkodine), intervention 2 (role-playing = Poshteh), control (Masseur) Were. Subjects were then randomly selected in each district.

Blinding (investigator's opinion)

Single blinded

Blinding description

In order to blind the participants, individuals in each of the intervention and control groups were selected from three separate marginal areas to avoid communication with each other and not to be informed about each other's education.

Placebo

Not used

Assignment

Parallel

Other design features

Secondary Ids

empty

Ethics committees

1

Ethics committee

Name of ethics committee

Institutional research ethics committee, Isfahan University of medical Sciences

Street address

hezarjarib

City

Isfahan

Province

Isfehan

Postal code

۷۳۴۶۱-۸۱۷۴۶

Approval date

2019-10-23, 1398/08/01

Ethics committee reference number

IR.MUI.RESEARCH.REC.1398.482

Health conditions studied

1

Description of health condition studied

HIV

ICD-10 code

Z71.7

ICD-10 code description

Human immunodeficiency virus [HIV] counseling

Primary outcomes

1

Description

knowledge

Timepoint

two weeks

Method of measurement

A standardized questionnaire assessing the knowledge and attitude of the general population regarding HIV

2

Description

attitude

Timepoint

two weeks

Method of measurement

A standardized questionnaire assessing the knowledge and attitude of the general population regarding HIV

Secondary outcomes

empty

Intervention groups

1

Description
"Intervention group1: pamphlets and lecture"; Four one-hour training sessions per week; One month training period.; The educational content will be prepared from the brochure "A New Approach to HIV Education", published by the Ministry of Health and Medical Education.
Category
Behavior

2

Description
"Intervention group 2: pamphlets and role-playing"; Four one-hour training sessions per week; One month training period.; The educational content will be prepared from the brochure "A New Approach to HIV Education", published by the Ministry of Health and Medical Education.
Category
Behavior

3

Description
No training will be provided except for a pamphlet.
Category
Behavior

Recruitment centers

1

Recruitment center
Name of recruitment center
Lorestan university of medical sciences
Full name of responsible person
Mina Jomezadeh
Street address
Moalem st
City
Khorram Abad
Province
Lorestan
Postal code
6813833946
Phone
+98 66 3330 0661
Email
publicrelation@lums.ac.ir

Sponsors / Funding sources

1

Sponsor
Name of organization / entity
Esfahan University of Medical Sciences
Full name of responsible person
Shaghayegh Haghjooy Javanmard
Street address
hezarjarib
City
Isfahan
Province
Isfehan
Postal code
۷۳۴۶۱-۸۱۷۴۶
Phone
+98 31 3792 3161
Email
maryamnasirian17@gmail.com
Grant name
Grant code / Reference number
Is the source of funding the same sponsor organization/entity?
No
Title of funding source
Deputy of Research and Technology in Isfahan University of Medical Sciences
Proportion provided by this source
100
Public or private sector
Public
Domestic or foreign origin
Domestic
Category of foreign source of funding
empty
Country of origin
Type of organization providing the funding
Academic

Person responsible for general inquiries

Contact
Name of organization / entity
Khoram-Abad University of Medical Sciences
Full name of responsible person
Mina Jomezadeh
Position
Manager of Health Workers Training Center
Latest degree
Master
Other areas of specialty/work
Health Promotion
Street address
Moalem
City
Khorram abad
Province
Lorestan
Postal code
6813833946
Phone
0986633232171
Email
pomin15@yahho.com

Person responsible for scientific inquiries

Contact
Name of organization / entity

Esfahan University of Medical Sciences
Full name of responsible person
Maryam Nasirian
Position
Faculty Member
Latest degree
Ph.D.
Other areas of specialty/work
Epidemiology
Street address
Hezarjarib
City
Esfahan
Province
Isfehan
Postal code
۷۳۴۶۱-۸۱۷۴۶
Phone
0983137923161
Email
Maryamnasirian17@gmail.com

Person responsible for updating data

Contact
Name of organization / entity
Esfahan University of Medical Sciences
Full name of responsible person
Maryam Nasirian
Position
Faculty Member
Latest degree
Ph.D.
Other areas of specialty/work
Epidemiology
Street address
Hezarjarib
City
Esfahan
Province
Isfehan
Postal code
۷۳۴۶۱-۸۱۷۴۶
Phone
+98 31 3668 0048
Email
Maryamnasirian17@gmail.com

Sharing plan

Deidentified Individual Participant Data Set (IPD)
No - There is not a plan to make this available
Justification/reason for indecision/not sharing IPD
confidential consideration about HIV issue and marginalized population
Study Protocol
No - There is not a plan to make this available
Statistical Analysis Plan
No - There is not a plan to make this available
Informed Consent Form
No - There is not a plan to make this available
Clinical Study Report
No - There is not a plan to make this available
Analytic Code
No - There is not a plan to make this available
Data Dictionary
No - There is not a plan to make this available
