# Supplementary material for: Improving HIV stigma in the marginalized population in Khorramabad, Iran: A single-blinded randomized, controlled educational trial using role-playing and lecturing
Source: PLOS Glob Public Health. 2023 Mar 7;3(3):e0000689. doi: 10.1371/journal.pgph.0000689 (PMC10022175; doi:10.1371/journal.pgph.0000689)
Supplement: Attachment — Submitted filename: Journal Requirements.docx [file pgph.0000689.s002.docx]

Journal Requirements:

1. Please provide a detailed online Financial Disclosure statement. This is published with the article. It must therefore be completed in full sentences and contain the exact wording you wish to be published.

a. Please clarify all sources of funding (financial or material support) for your study. List the grants (with grant number) or organizations (with url) that supported your study, including funding received from your institution. 
b. State the initials, alongside each funding source, of each author to receive each grant.
c. State what role the funders took in the study. If the funders had no role in your study, please state: “The funders had no role in study design, data collection and analysis, decision to publish, or preparation of the manuscript.”
d. If any authors received a salary from any of your funders, please state which authors and which funders.

If you did not receive any funding for this study, please simply state: “The authors received no specific funding for this work.”

*Thanks for this comment. As we did not receive any funding for this study, we add “The authors received no specific funding for this work.” in the manuscript.*

2. Please update your online Competing Interests statement. If you have no competing interests to declare, please state: “The authors have declared that no competing interests exist.”

*Thanks for this comment. As we had no competing interests, we add “The authors have declared that no competing interests exist.” in the manuscript.*

3. Please provide separate figure file in .tif or .eps format and ensure that all files are under our size limit of 10MB.

*Thanks for this comment. The mentioned format was applied.*

For more information about how to convert your figure files please see our guidelines: <https://journals.plos.org/globalpublichealth/s/figures>

4. We have noticed that you have uploaded Supporting Information files, but you have not included a list of legends. Please add a full list of legends for your Supporting Information files after the references list.

*Thanks for this comment.we add list of legend in the manuscript end.*

**Proofs corrections**

| Journal  **PLOS Global Public Health** |  |
| --- | --- |
| Article title  **Improving the HIV-stigma in the Marginalized population in Khorramabad, Iran: a single-blinded randomized, controlled educational trial using role-playing and lecturing** |  |
| I would like to recheck the corrections: Yes |  |

If you have access to Acrobat, it may be helpful to mark the corrections in the PDF file using PENCIL and NOTE tools. Alternatively provide the list of corrections using this table. Please make the corrections’ list self-explanatory and easy to understandable for a non-medical technical person.

**List of corrections in the first revision**

| **Reviewer Number** | **Original comments of the reviewer** | **Reply by the author(s)** | **Changes done on page number and line number** |
| --- | --- | --- | --- |
| Reviewer 1 | - The authors wrote: "According to the report of the Ministry of Health, there were 41494 people infected with HIV by the end of 2019, and deaths of 19164 people were recorded [1]." It is unclear what Ministry of Health the authors are referring to. Following this, I suggest that the authors should make mention of what country their study is based on because the next sentence goes to mention a province. Not all the readers would be privy with such information and so the authors should consider providing it. | Thanks for your valuable comment. The sentences were revised. | Introduction, the first paragraph, line 3 |
| Reviewer 1 | - Furthermore, there were about 1293 cases I n Lorestan province, but based on a study by Poorolajal et al. (2017), the number of people living with HIV in the capital of this province, Khorramabad, is probably much higher and is estimated at 2456 ]2[, and it seems that some of them are residents of three marginalized regions around Khorramabad, but unfortunately there is no accurate information about the number of patients in these regions.----This is a very long sentence, the authors should consider breaking this to two or three sentences. | Thanks for your valuable comment. The sentences were revised. | Introduction, the first paragraph, line 4-6. |
| Reviewer 1 | - Residents of marginalized regions are vulnerable populations to HIV ---- It is unclear what the authors mean by marginalised regions in this context. | *Thanks for your valuable comment. The sentences was revised.* | *Introduction, the second paragraph, line 1.* |
| Reviewer 1 | - The authors wrote: HIV/AIDS has become an acute problem in the health system worldwide ---- I am not sure if this is accurate. Is HIV an "acute problem". I thought it is more than endemic? The authors should consider reviewing the entire sentence for accuracy. | Thanks for your valuable comment. The sentences was corrected. | Introduction, line 15. |
| Reviewer 1 | -The authors wrote: "The World Health Organization's main activities seem to focus on treating the patients and preventing infection by controlling the ways of its transmission…" ---- This is not completely accurate; it is only recently with findings showing that undetectable viral load tremendously reduces the chances of HIV transmission. But before this and even up to now there are still predominant HIV prevention methods including most recently the use of PrEP. | *Thanks for your valuable comment. The sentences were revised.* | *Introduction, the third paragraph, line 2-3.* |
| Reviewer 1 | -According to statistics in Iran, about 15.4% of HIV-infected people do not go to service centers to receive services ---- The authors should consider citing this source. | *for your valuable comment. The reference was added.* | *Introduction, the third paragraph, line8.* |
| Reviewer 1 | It is unclear to me the overall population from which the sample was obtained. I know the authors used the power calculation based on the three arms but what is the estimated population from which the sample was drawn. | Thanks for your valuable comment. We added the total population and separately in three regions. | The fourth paragraph, In sampling. line 4-5.  In addition, we revised the figure1. |
| Reviewer 1 | - We included a total of 270 individuals (51% women) aged 18 to 49 years in marginalized regions, Falak od Din (role playing: 80), Masur (Control: 1100) and Poshteh (Lecture: 80) ---- I think that there is a typo on the Control group number. Is that supposed to be 110? | Thank you for your very good and accurate comment.  *The number of 1100 was revised to 110*. | Results, the first paragraph, line2 |
| Reviewer 1 | - The mean score of knowledge was significantly lower in the control group before the intervention than the intervention groups (p=0.001) ---- This result is not too clear to me especially the phrase "before the intervention than the intervention groups". | *Thanks for your valuable comment. The sentences was revised.* | *Results, The second paragraph, line2-3.* |
| Reviewer 1 | - Despite the lack of difference between the effectiveness of role-playing and lecture in improving attitude in the present study, studies have found that role-playing had a greater effect on improving knowledge and attitude than lecture, for instance, Manzari et al. (2015) found that more advanced training methods such as role-playing and multimedia could be more effective than the lecture in promoting knowledge and attitude ]19 ,18[. ---This sentence contain two referencing styles. | *Thanks for your valuable comment. The sentences were revised.* | *Discussion, the third paragraph, line 5-8.* |
